# Increase in complement iC3b is associated with anti-inflammatory cytokine expression during late pregnancy in mice

**DOI:** 10.1371/journal.pone.0178442

**Published:** 2017-05-25

**Authors:** Keigo Nakamura, Kazuya Kusama, Rulan Bai, Sadamasa Ishikawa, Sayuri Fukushima, Yoshihito Suda, Kazuhiko Imakawa

**Affiliations:** 1 Animal Resource Science Center, Graduate School of Agricultural and Life Sciences, The University of Tokyo, Kasama, Ibaraki, Japan; 2 Department of Food, Agriculture and Environment, Miyagi University, Taihaku, Sendai, Miyagi, Japan; Colorado State University College of Veterinary Medicine and Biomedical Sciences, UNITED STATES

## Abstract

Immunological tolerance between fetal allograft and mother is crucial for pregnancy establishment and maintenance; however, these mechanisms particularly those during the latter part of pregnancy have not been definitively elucidated. The aim of this study was to examine the presence and potential function of innate immunity characteristic to the middle to late pregnancy. We first characterized up-regulated proteins in decidua from day 11 pregnant (P11) mice using 2D-PAGE, followed by MALDI-TOF/MS analysis. These analyses identified increased complement component 3 (C3) and its derivatives in P11 decidua. We then found that in the decidual tissues, *C3* mRNA increased on P15 and remained high on P19. C3 is converted to C3b and then iC3b by complement component factor I (*Cfi*) and complement receptor 1-like protein (*Crry*), both of which were present in P19 placentas. In addition, iC3b proteins and its receptor CR3 (*Cd11b/Cd18*) in decidual and placental tissues increased toward the latter phase of pregnancy. Moreover, CR3 subunit CD11b protein was predominantly localized to spongiotrophoblast layer in the P19 placenta. Because iC3b is known to induce anti-inflammatory cytokine production, the analysis was extended to examine changes in pro- and anti-inflammatory cytokines, *Il12*, *Il10*, and *Tgfb1*. *Il12* expression decreased in P15 and P19 placenta, while high mRNA expression of *Il10* and *Tgfb1* was found in P19 placental tissues. Furthermore, placental *Il10* and *Tgfb1* mRNAs were down-regulated when pregnant mice were treated with an anti-C3 antibody, detecting C3, C3b and iC3b. These results indicated that C3 derivatives, in particular, iC3b and its receptor CR3 were up-regulated at the fetal-maternal interface, and suggest that iC3b may regulate the placental expression of anti-inflammatory cytokines, IL10 and TGFB1, during the latter phase of pregnancy.

## Introduction

Successful placentation and subsequent maintenance of pregnancy requires an intimate immunological relationship between mother and fetus [1]; however, these processes face a challenge because the fetus and placenta carry paternal genes allogenic to the maternal immune system. The semi-allogeneic fetus and the mother must establish complex immune systems in pregnancy, by which the fetal allograft is protected while invaders such as bacteria and other microorganisms are eliminated. With this immunological paradox in mind, various studies on both innate and adaptive immune systems have been conducted over the last five decades [2–8]. In fact, researches related to adaptive immunity have been focused, but importance and/or contribution of innate immunity on pregnancy immunology is often overlooked.

The complement system, a major contributor to the innate immunity, is composed of more than 30 proteins, of which cascades are activated by classical, lectin, and/or alternative pathways [9], resulting in the generation of complement component 3 (C3) [10]. In the alternative pathway, C3 is hydrolyzed to C3_H2O_ and to form C3 convertase with complement factor B (CFB) and complement factor D (CFD), generating active fragments, C3a and C3b [11, 12]. Whereas C3a plays a role of anaphylatoxins, facilitating pathogen clearance, inducing inflammatory cell chemotaxis, and releasing cytokines [10], C3b functions with CFB and CFD to generate more C3b depositions which promote opsonization, migration of phagocytes, and cell lysis. C3b is then cleaved by complement component factor I (CFI) to generate inactive C3b (iC3b) and this process is mediated by complement receptor-1 (CR1, CD35), membrane cofactor protein (MCP), complement component factor H (CFH) and complement receptor 1-like protein (CRRY) [13, 14]. Furthermore, in the anterior chamber of the eye, iC3b binding to complement receptor type 3 (CR3) is essential for anti-inflammatory cytokine production, resulting in the induction of tolerance [15]. These findings indicate that in addition to known functions such as opsonization and anaphylatoxins classically characterized, complement systems have multiple functions, including the regulation of cytokine productions.

Complement components are primarily synthesized by the liver, but can also be synthesized locally in numerous tissues including placenta [16]. It was reported that the activated C3 may assist in trophoblast invasion of the decidua and endometrial blood vessels [17]. In *C3* or *C1q* deficient mice, reduced placental development in the labyrinth is demonstrated, suggesting that breakdown in the complement system may lead to abnormal placentation and pregnancy complications [18, 19]. Moreover, C1q and C9 are found in normal placentas, which lack in the placenta of preeclampsia patients [20]. Data accumulated thus far indicates that a functional complement system is required for not only maintenance of host defense but also successful embryo implantation and placental development. Despite advances in the understanding of the complement components, the exact role of complement system at the fetal-maternal interface, especially from placentation to pregnancy maintenance or latter part of pregnant period, has not been well studied.

We therefore hypothesized that in addition to adaptive immunity, the innate immunity characteristic to the middle to late pregnancy should be functioning, maintaining fine balance between pro- and anti-inflammatory environments. To test this hypothesis, we first characterized innate immunity related proteins in decidual tissues from day 11 pregnant mice using two dimensional-Polyacrylamide Gel Electrophoresis (2D-PAGE), followed by matrix assisted laser desorption ionization time-of-flight mass spectrometry (MALDI-TOF/MS). From the results of MALDI-TOF/MS analysis, we further investigated the identification, expression, localization and a potential role of complement systems at the fetal-maternal interface during the latter part of pregnancy.

## Materials and methods

### Animals and tissue preparation

Adult ICR mice (Kumagai-Shigeyasu Co. Ltd., Miyagi, Japan) were housed in a temperature- and light-controlled room (12 h light, 12 h dark cycle) and allowed free access to food and water. Timed mating of animals was conducted by placing females (>6 weeks of age) with fertile males or vasectomized males (day 1, vaginal plug), the latter treatment was for the generation of pseudopregnant mice. Subsequently, mice were killed and hysterectomy was performed on day 11 of pseudopregnancy (n = 3) or days 11, 15, 17, and 19 of pregnancy (P11, P15, P17, and P19, respectively; n = 3 each day). After hysterectomy, endometria, decidua and placentas were excised from uterine tissues. In addition, a female adult goat (40 months of age), maintained at Miyagi University farm, was used as the source of erythrocytes. To elute possibly adsorbed serum proteins, goat red blood cells (gRBC) collected from the carotid artery were centrifuged at 2000 × g for 5 min at 4°C three times and diluted 1:20 with PBS (5% gRBC). The concentration was photometrically adjusted to 1.5 x 10^8^ cells/ml.

All animal care and surgical procedures were approved by the Institutional Animal Care Committees at Miyagi University (approval numbers 2015–17 and 2017–02), in compliance with institutional guidelines for the care of experimental animals.

### 2D-PAGE

Endometrial tissues from day 11 pseudopregnant (n = 3) or decidual tissues from P11 mice (n = 3) were dissolved in 90 μl urea buffer (0.06 M Tris hydroxymethyl aminomethane, 1 M thiourea, 6 M urea, 3% CHAPS, 1% Triton X-100) and centrifuged at 15,000 × g for 30 min at 4°C. Supernatant was recovered and protein concentrations were measured using Benchmark Plus microplate spectrophotometer (Bio-Rad Laboratories, Inc., Hercules, CA) and adjusted to 1 μg/μl.

After measuring the protein concentrations, tissue lysates (10 μg) were mixed with 1 M acrylamide solution and were separated in agar gel (pH range: 3–10) (ATTO, Tokyo, Japan) and 5–20% SDS-polyacrylamide gradient gel (ATTO) according to the protocol provided by the manufacturer. After 2D-PAGE, the gel was stained with SYPRO Ruby (Thermo Fisher Scientific, Inc., Waltham, MA) overnight and washed with distilled water. The images were then captured using an LAS-3000 camera (FUJIFILM, Tokyo, Japan).

### Analysis by MALDI-TOF/MS

Proteins especially up-regulated in P11 deciduas were identified by MALDI-TOF/MS (Applied Biosystems, Foster City, CA). The protein spot in SYPRO Ruby-stained gel was manually excised, decolorized, dehydrated, and whitened according to the instruction and operation manual. The dried protein spot was digested with 0.01 μg/μl trypsin at 35°C overnight. The digested peptides and HCCA solution (10 mg/ml α-cyano-4-hydroxy-cinnamic acid (HCCA), 70% methanol, and 0.1% trifluoroacetic acid) were orderly placed on an MTP AnchorChip 800/384 target. Protein identification was conducted by MALDI-TOF/MS. Peptide standard with a mass range of 700–3200 Da was used as external calibration. The parameters of MALDI-TOF/MS were set as follows: reflection mode, protein mass m/z 800–4000, trypsin as the digestion enzyme with one miss-cleavage site, fixed modifications carbamidomethyl (C), variable modifications oxidation (M), peptide mass tolerance ± 100 ppm, fragment mass tolerance ± 0.5 Da, number of matched peptide fragments at least 3 pieces, and mass spectral peaks m/z ≥ 600.

### RNA extraction and quantitative RT-PCR

Using the ISOGEN reagent (Nippon Gene, Tokyo, Japan), total RNAs were extracted from days 11, 15, 17, and 19 deciduas and placentas (n = 3 each day) according to the manufacturer’s protocol. For quantitative real-time PCR (qPCR) analyses, isolated RNA (total 250 ng) was reverse-transcribed to cDNA using ReverTra Ace qRNA RT Kit (Toyobo, Osaka, Japan), and the resulting cDNA (RT template) was stored at 4°C until use [21].

Reverse-transcribed cDNA was subjected to qPCR amplification using Thunderbird SYBR qPCR Mix Kit (Toyobo) with 0.3 μM of the oligonucleotide primers listed in S1 Table, and qPCR amplification was carried out on an Applied Biosystems STEP One Plus real-time PCR System (Applied Biosystems) [22]. Amplification efficiencies of each target gene and two reference genes, actin-beta (*Actb*) and glyceraldehyde 3-phosphate dehydrogenase (*Gapdh*), were examined through their calibration curves and found to be comparable. The thermal profile for qPCR consisted of 40 cycles at 95°C for 10 sec and annealing and extension at 60°C for 45 sec. Average threshold (Ct) values for each target were determined by Sequence Detection System software v2.2 (Applied Biosystems). Each run was completed with melting curve analysis to confirm the specificity of amplification and the absence of primer dimer.

### Western blot analysis

To determine expression of C3, C3b, and iC3b, decidual or placental tissues (n = 3 each day) were prepared in lysis buffer (50 mM Tris-HCl, 150 mM NaCl, 1 mM ethylenediaminetetraacetic acid (EDTA), 1% Triton X-100, 0.1% sodium dodecyl sulfate, 1 mM Na_3_VO_4_, and 50 mM NaF). Decidual and placental tissue lysates (10 μg/lane) were separated through 12.5% SDS-PAGE and were then transferred onto a total of three polyvinylidene difluoride (PVDF) membranes (Millipore, Milford, MA). After blocking with Block Ace reagent (DS Pharma Biomedical, Osaka, Japan), membranes were incubated with rabbit monoclonal anti-human C3 antibody (1:2000, ab200999, Abcam, Tokyo, Japan), rabbit polyclonal anti-mouse CD11b antibody (1:500 dilution, 0.5 mg/ml, ab75476, Abcam), or rabbit monoclonal anti-human ACTB antibody (for internal control, 1:1000, ab1801, Abcam). Immunoreactive bands were detected using enhanced chemiluminescence (Millipore) after incubation with horseradish peroxidase-labeled SAP solution (APRO life Science, Inc., Tokushima, Japan).

### Immunohistochemistry

Immunohistochemical analysis was performed on 10 μm fresh-frozen sections of P19 placental tissue blocks (n = 3 animals). Frozen sections were fixed with 4% paraformaldehyde/PBS, and endogenous peroxidase was quenched by immersing in 0.3% (v/v) hydrogen peroxide/methanol. A streptavidin/biotin blocking kit (Vector Laboratories, Burlingame, CA) was used to block endogenous biotin according to the manufacturer's instructions. After 30 min of incubation with 10% normal goat serum, the sections were incubated at 4°C overnight with a rabbit anti-mouse CD11b polyclonal antibody (1:100 dilution, 0.5 mg/ml, ab75476, Abcam), a rabbit anti-mouse trophoblast specific protein alpha (TPBPA) antibody (1:100 dilution, 1 mg/ml, ab104401, Abcam), or a normal rabbit IgG (1:40 dilution, 0.4 mg/ml, sc-2027, Santa Cruz Biotechnology, Inc., Dallas, TX) as a negative control. Subsequently, the sections were incubated at room temperature for 1 h with a goat anti-rabbit IgG biotin conjugate (1:800 dilution, B8895, Sigma–Aldrich, St. Louis, MO). The immunoreactivity was visualized using the avidin-peroxidase (1:400 dilution, E2886, Sigma-Aldrich) and AEC substrate kit (Thermo Fisher Scientific, Inc., Waltham, MA) according to the manufacturer's instructions and then examined under light microscope (BX-51, Olympus, Tokyo, Japan).

### Antibody neutralization study

To further study the association between iC3b protein and the expression of anti-inflammatory cytokines, anti-C3 antibody (500 μg/sample, 8.9 mg/ml, 204969, Millipore), which neutralizes C3, C3b, and iC3b, or PBS was injected into the tail vein using a 30-gauge needle on P15. 48 hours following the anti-C3 antibody injection, decidual or placental tissues were dissected on P17 (n = 3), from which RNA was extracted and subjected to qPCR analysis for anti-inflammatory mRNA expression. The blood samples were collected by venous puncture in sterile tube and centrifuged within 1 hour of collection, from which serum for hemolytic complement test was stored at -80°C until use.

### Alternative hemolytic complement test

The alternative hemolytic complement test was performed using goat erythrocytes (gRBC). Mice serum samples (n = 3) diluted 1:10 in Gelatin Veronal Buffer with Mg and Eethylene glycol tetraacetic acid (EGTA) (5 mM Barbital, 145 mM NaCl, 0.5 mM MgCl_2_, 10 mM EGTA, 0.1% Gelatin) were incubated with 5% gRBC/PBS at 37°C for 40 min. Gelatin Veronal Buffer with Mg and EDTA (10 mM Barbital, 145 mM NaCl, 10 mM EDTA, 0.1% Gelatin) was added to stop the reaction and the mixtures were centrifuged at 2,000 × g for 10 min at 4°C. The extent of hemolysis was determined by reading the optical density of the supernatant at 413 nm. Degree of lysis was calculated relative to the 100% lysis control (erythrocyte suspension and distilled water).

### Statistical analysis

Data were expressed as the mean ± SEM. Significance was assessed using t-test or the Tukey-Kramer test. A *P*-value < 0.05 was considered statistically significant.

## Results

### Up-regulated proteins in decidual tissues from Day 11 pregnant mice

**Fig 1:**
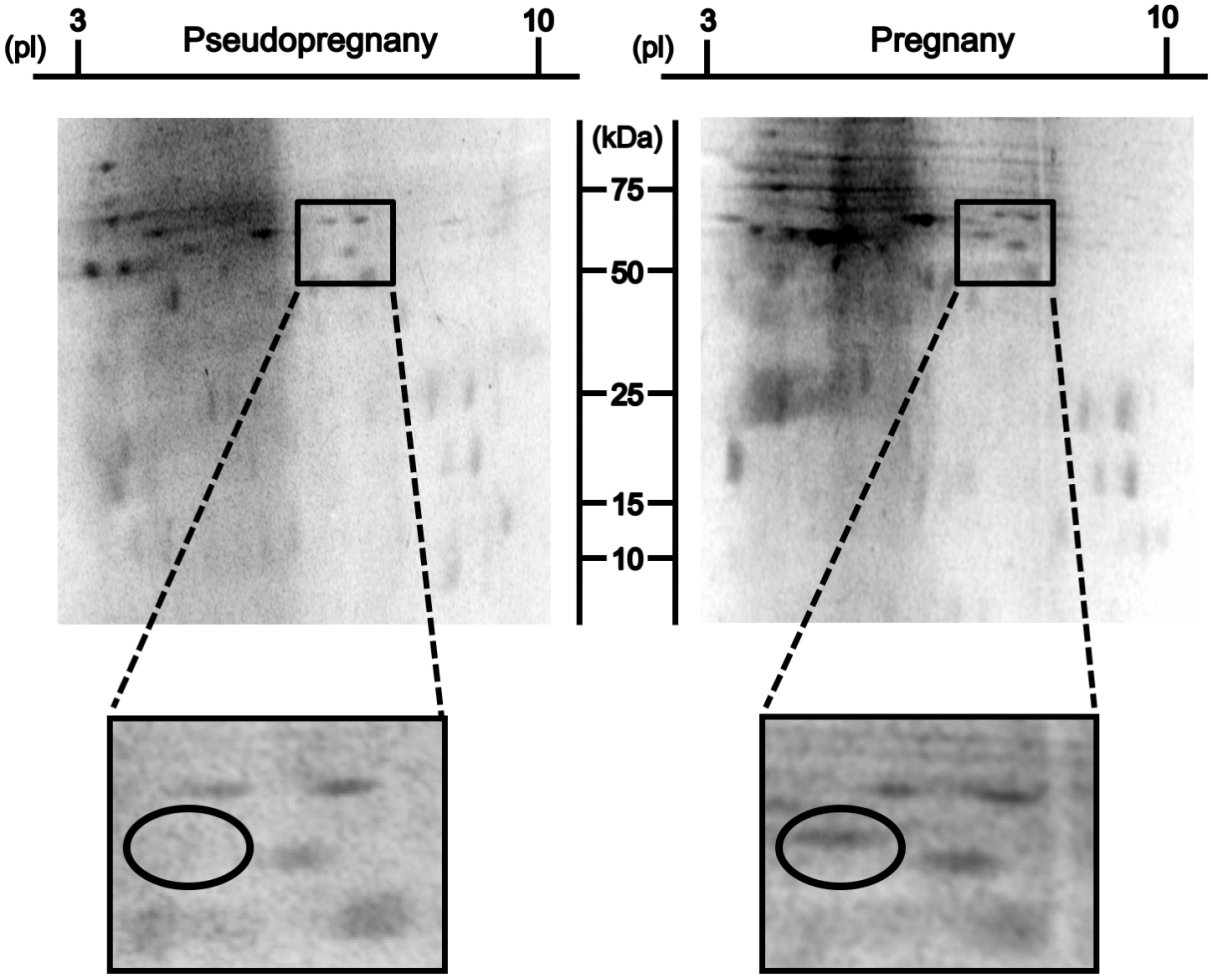
Up-regulated proteins in P11 decidual tissues. SYPRO Ruby stained 2D-PAGE gel images of endometrial tissue from day 11 pseudopregnant (Left panel) mice and decidual tissue from P11 (Right panel) mice. Proteins in the circled spot in P11 decidual tissues were up-regulated compared to those in day 11 pseudopregnant endometrial tissues, which were subjected to MALDI-TOF/MS analysis. Protein migration patterns were reproducible from animal to animal, and a representative 2D gel from three independent experiments is shown.

Results from 2D-PAGE revealed that although similar protein migration patterns between pseudopregnant endometrial tissues and P11 decidual tissues were recognized, P11 decidual tissues contained increased proteins compared to those from the pseudopregnant endometrial tissues (Fig 1). Spots especially up-regulated in P11 decidual tissues were cut off and subjected to MALDI-TOF/MS analysis, which revealed that a total 45 peptides were identified from P11 up-regulated spots (S2 Table). These decidual peptides were each subjected to BLAST search, identifying C3, and its derivatives C3b and iC3b proteins.

### *C3* mRNA and C3, C3b, and iC3b protein expression in decidual and placental tissues from middle to late pregnancy

**Fig 2:**
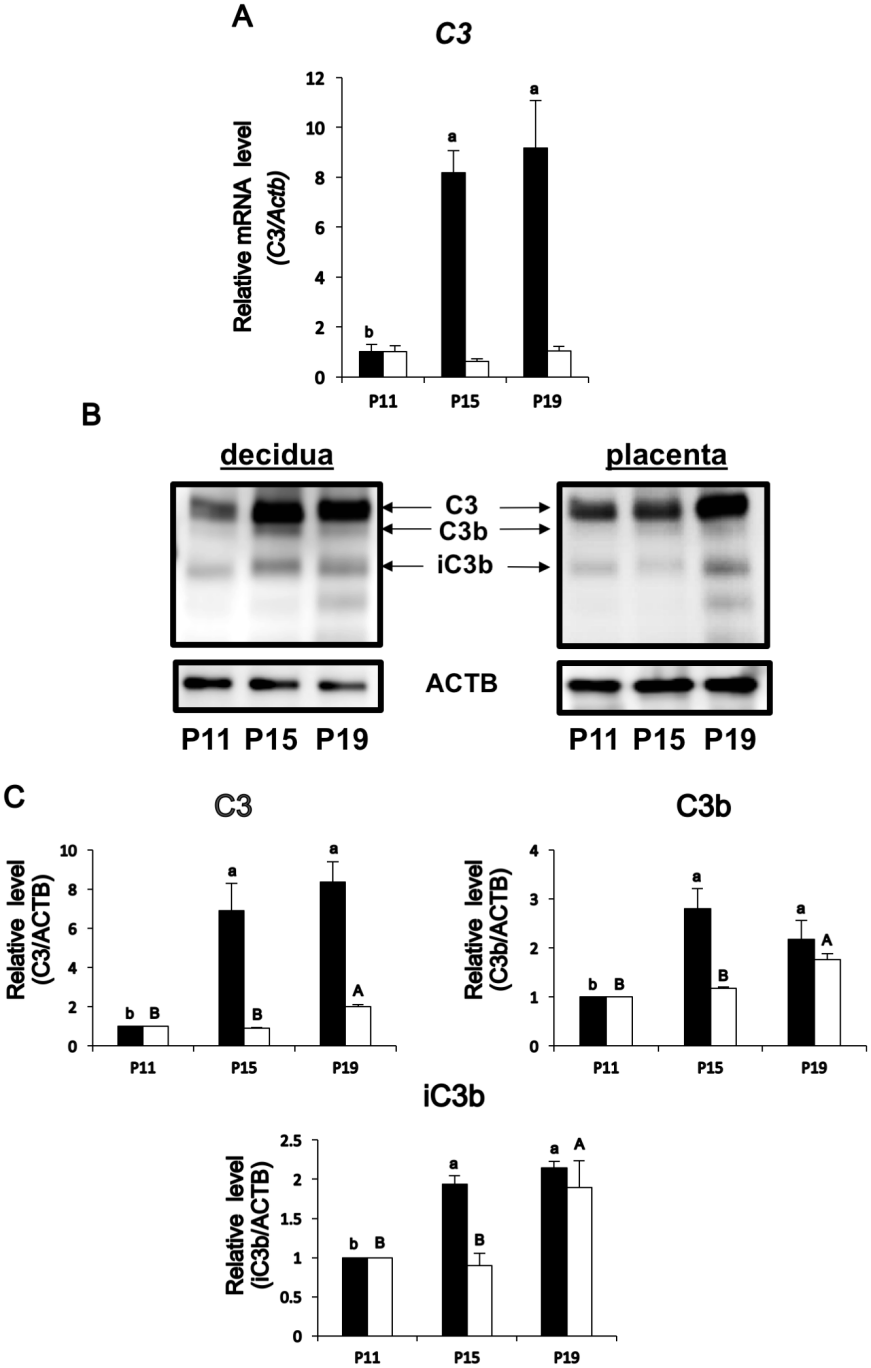
Expression of C3 mRNA and C3, C3b, and iC3b proteins in the decidual and placental tissues. (A) Levels of *C3* mRNAs obtained from P11, P15, or P19 decidua (solid bar) and P11, P15, or P19 placenta (white bar) (n = 3 mice each day). *Actb* mRNA was used as an internal control for RNA integrity. Values represent mean ± SEM from three animals with duplicate samples. Different superscripts indicate statistically significant differences in *C3* mRNA levels (p < 0.05) when compared to that of P11. (B) Western blot analysis of C3, C3b, or iC3b expression in P11, P15, or P19 decidual and P11, P15, or P19 placental tissues. ACTB was used as an internal control. A representative data from three independent experiments containing protein samples from three different animals is shown. (C) Densitometric quantitation of C3, C3b, and iC3b on Western blot analysis. The analysis was done using LI-COR C-DiGit Blot Scanner and the relative density of the blots was determined by normalization against ACTB levels. Values represent mean ± SEM from three animals with duplicate samples. Different superscripts (small letters, decidua) or capital letters (placenta) indicate statistically significant differences in mRNA levels (p < 0.05) within the tissues when compared to that of P11.

To further investigate the results from MALDI-TOF/MS analysis, qPCR analysis for *C3* and western blot analysis for C3, and its derivatives, C3b and iC3b, were carried out in the decidual and placental tissues. *C3* mRNA levels were increased by approximately eight- to nine-fold (vs P11) in P15 and P19 decidual tissues, respectively, but those of placental tissues were not changed on these days (Fig 2A). Western blot analysis then revealed that C3, C3b, and iC3b proteins were detected. C3 protein expression increased in P15 and P19 decidual tissues, but not in the placenta (Fig 2B and 2C). C3b expression was peaked on P15 but decreased on P19 in the decidua, whereas iC3b expression level was increased in P19 placenta. Moreover, a degradative product of iC3b, C3c, expression was also high in P19 decidual and placental tissues (data not shown).

### High expression of iC3b in uterine decidua and placenta in late pregnancy

**Fig 3:**
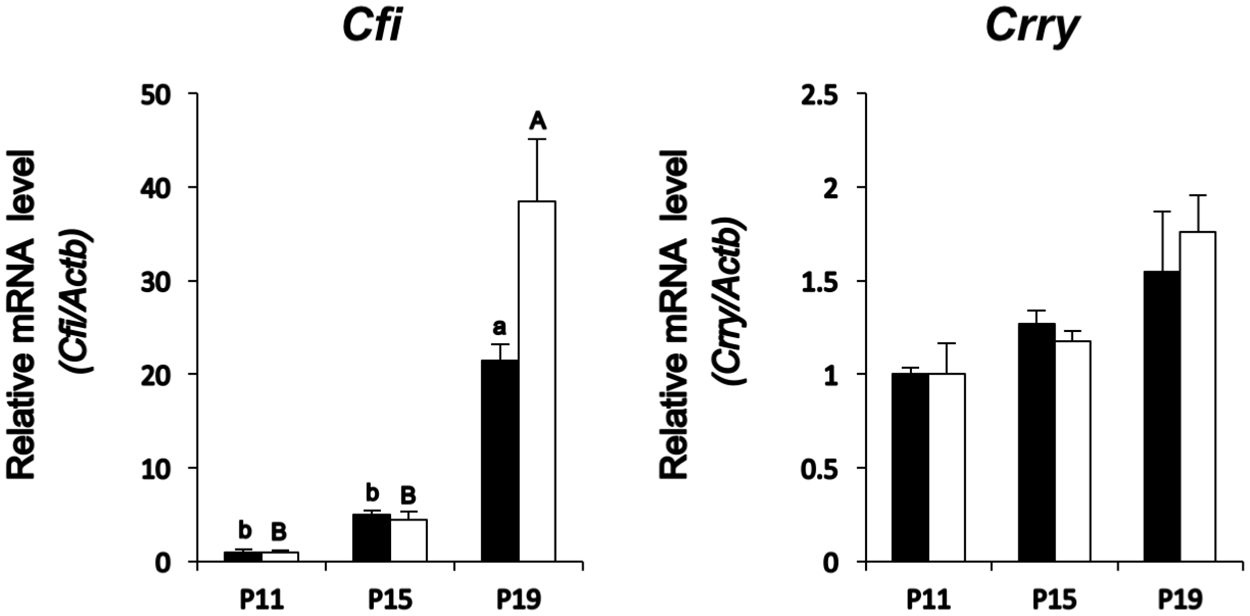
Expression of C3b regulator, Cfi, and its cofactor, Crry, in the uterine decidua and placenta. Changes in *Cfi* (Left panel) and *Crry* (Right panel) mRNAs in P11, P15, or P19 decidua (solid bar) and P11, P15, or P19 placenta (white bar) (n = 3 mice each day). *Actb* mRNA was used as an internal control for RNA integrity. Values represent mean ± SEM from three animals with duplicate samples. Different superscripts or letters indicate statistically significant differences in mRNA levels (p < 0.05) when compared to that of P11.

Because C3b is converted to iC3b through the expression of C3b regulator CFI and its cofactor CRRY [23], qPCR was executed to determine abundance of *Cfi* and *Crry* mRNA in RNA extracted from P11, P15 or P19 decidual and placental tissues. *Cfi* expression was increased by twenty-fold and forty-fold in P19 decidua and P19 placenta, respectively (Fig 3A), whereas *Crry* transcript was expressed in both P19 decidual and placental tissues (Fig 3B). These results together with iC3b protein expression (Fig 2B and 2C) indicated that C3b was highly converted to iC3b by CFI in the presence of CRRY on P19 during the period just before parturition in mice.

### Up-regulated iC3b receptor expression in decidua and spongiotrophoblast layer of the placenta

**Fig 4:**
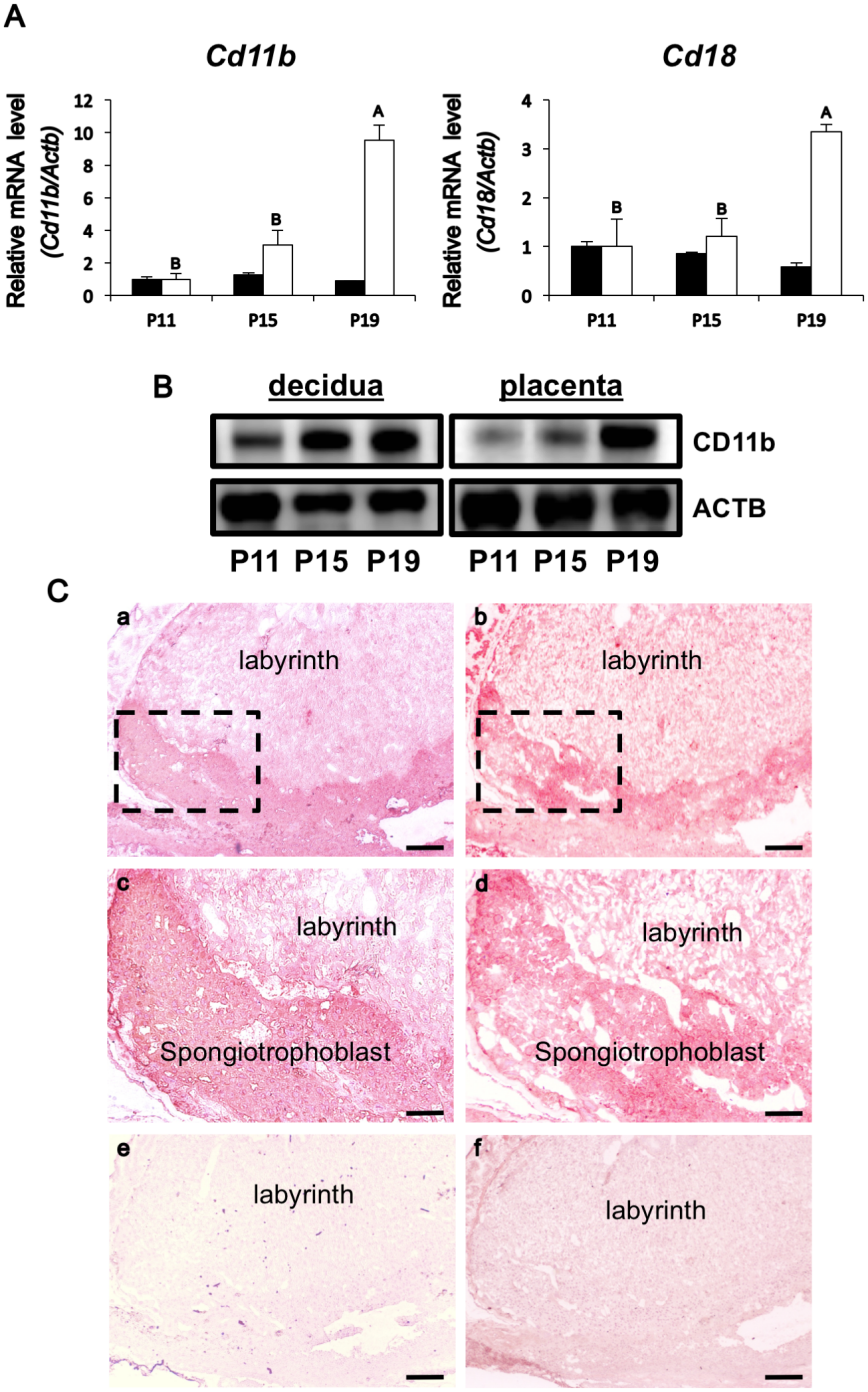
Expression and localization of iC3b receptor CR3 in the uterine decidua and placenta. (A) Changes in *CR3* (*Cd11b* or *Cd18*) mRNA levels in P11, P15, or P19 decidua (solid bar) and P11, P15, or P19 placenta (white bar) (n = 3 mice each day). *Actb* mRNA was used as an internal control for RNA integrity. Values represent the mean ± SEM from three animals with duplicate samples. Different letters indicate statistically significant differences in mRNA levels (p < 0.05) when compared to that of P11. (B) Western blot analysis of CD11b expression in P11, P15, or P19 decidual and P11, P15, or P19 placental tissues. ACTB was used as an internal control. A representative data from three independent experiments containing protein samples from three different animals is shown. (C) Immunohistochemical detection of TPBPA and CD11b in P19 mouse placenta. Tissue sections were immunostained for a spongiotrophoblast specific TPBPA (a, c, and e) using anti-TPBPA antibody (a and c) or normal rabbit IgG (e) as a negative control. Boxed area in (a) is shown at a higher magnification in (c). Tissue sections were immunostained for iC3b receptor subunit CD11b (b, d, and f) using anti-CD11b antibody (b and d) or normal rabbit IgG (f) as a negative control. Boxed area in (b) is shown at a higher magnification (100 x) in (d). Scale bar = 200 μm (a, b, e, and f), or 100 μm (c and d), respectively. A representative immunostaining from three independent experiments is shown.

Examination was extended to determine expression of iC3b receptor, CR3, composed of integrin αM (ITGAM, CD11b) and integrin β2 (ITGB2, CD18). qPCR analyses in RNAs extracted from uterine decidua and placenta on P11, P15 or P19 revealed that *Cd11b* and *Cd18* expressions were minimal in P11 placenta, but the P19 placenta exhibited a ten-fold increase in *Cd11b* expression and a three-fold increase in *Cd18* (Fig 4A). Although decidual *Cd11b* and *Cd18* mRNA expression did not differ, steady levels of these mRNAs were present in P11, P15 and P19 deciduas (Fig 4A). Western blot analysis revealed that similar to *Cd11b* transcript, CD11b protein expression was steady in deciduas, and up-regulated in P19 placental tissues (Fig 4B). To localize CD11b protein, immunohistochemical analysis was carried out with fresh-frozen sections from P19 placental tissues. The results revealed that the staining of CD11b was consistent with that of TPBPA, a protein marker specific to spongiotrophoblast layer [24, 25], indicating that iC3b receptor, CD11b, was localized to the spongiotrophoblast layer in the P19 placenta (Fig 4C).

### High expression of anti-inflammatory cytokines in late pregnancy

**Fig 5:**
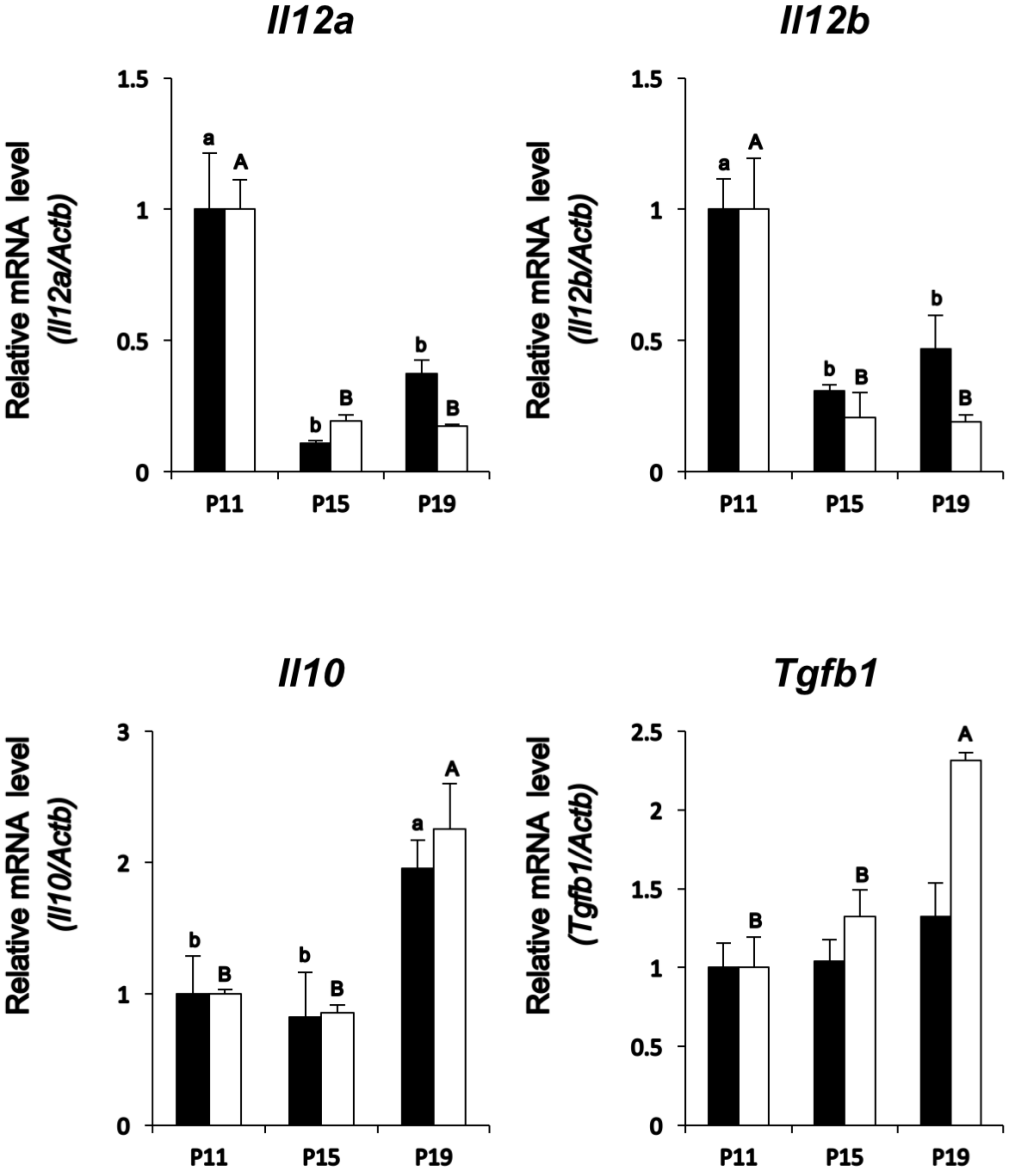
Expression of pro- and anti-inflammatory cytokines, *Il12*, *Il10*, and *Tgfb1*. Changes in *Il12a*, *Il12b*, *Il10*, or *Tgfb1* mRNAs in P11, P15, or P19 decidua (solid bar) and P11, P15, or P19 placenta (white bar) (n = 3 each day). *Actb* mRNA was used as an internal control for RNA integrity. Values represent the mean ± SEM from three animals with duplicate samples. Different small or capital letters indicate statistically significant differences in mRNA levels (p < 0.05) when compared to that of P11.

qPCR was executed to examine changes in transcript levels of pro-inflammatory cytokine, interleukin 12 (*Il12)*, composed of *Il12a* and *Il12b* subunits, and anti-inflammatory cytokines, interleukin 10 (*Il10*) and transforming growth factor beta 1 (*Tgfb1*), expression in P11, P15 or P19 decidual and placental tissues. *Il12a* and *Il12b* expression levels were decreased in P15 and P19 deciduas and placentas, while *Il10* transcript was highly expressed in P19 decidual and placental tissues and *Tgfb1* expression level was increased in P19 placenta (Fig 5). These changes indicated that rather than pro-inflammatory cytokine expression, the iC3b and its receptor CR3 are associated with the increase in anti-inflammatory cytokine expression in decidua and placenta from middle to late pregnancy.

### Down-regulation of anti-inflammatory cytokine expression with anti-C3 antibody in decidua and placenta

**Fig 6:**
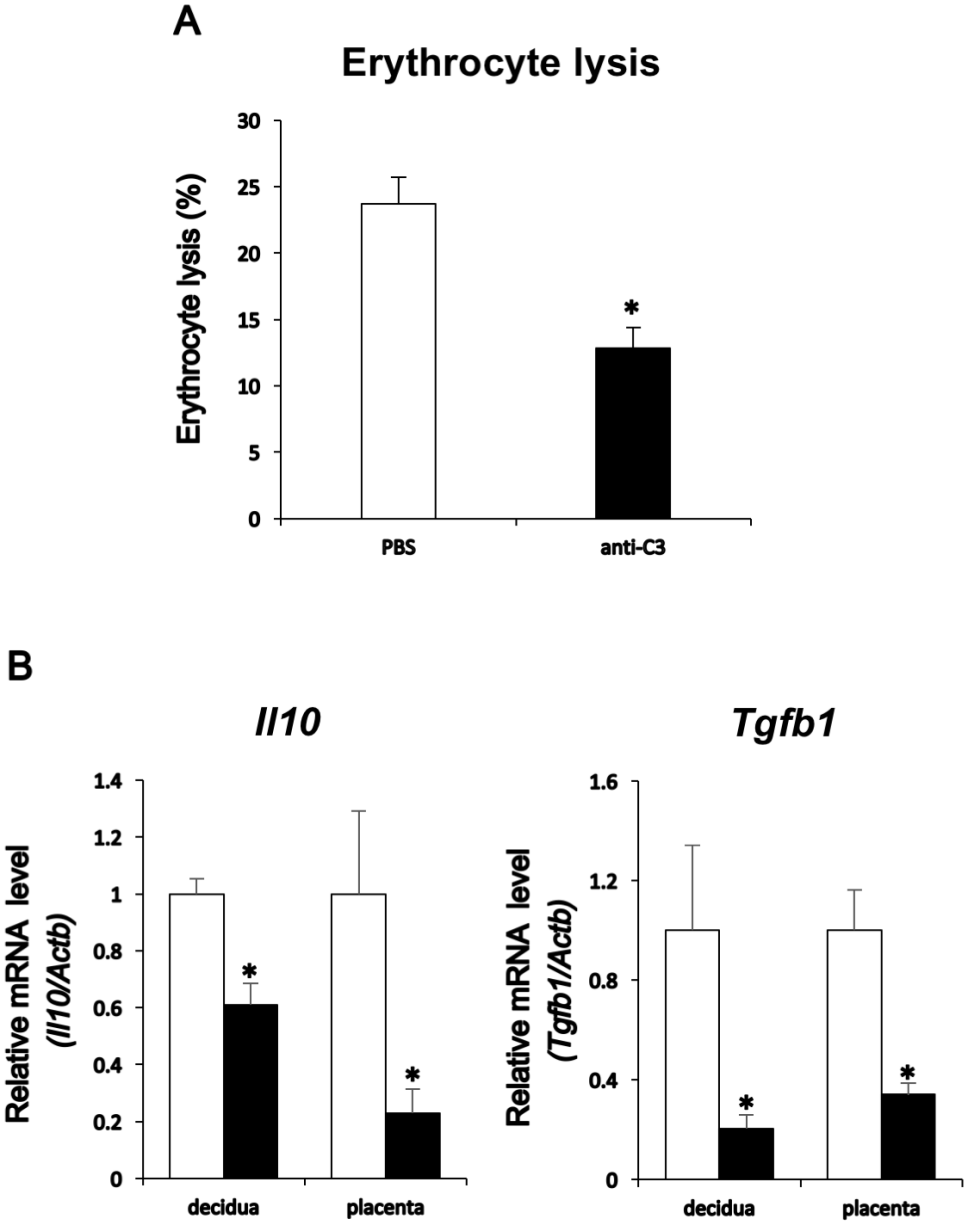
Down-regulation of anti-inflammatory cytokines, *Il10* and *Tgfb1*, by anti-C3 antibody. (A) Goat erythrocyte lysis against mice serum. PBS (white bar) for control or anti-C3 antibody (solid bar) was injected into the tail vein on day 15. 48 h following the injection, serum was recovered from caudal vein and reacted with 5% goat erythrocytes/PBS. Values represent mean ± SEM from three independent experiments, each containing duplicates. Asterisks indicate significant differences in erythrocyte lysis (P<0.05) when compared to that of the control, PBS treatment. (B) Effects of C3 neutralizing antibody, which detects C3, C3b, and iC3b, on the expression of anti-inflammatory cytokine transcripts in pregnant decidua and placenta. After 48 h from injection of PBS (white bar) or anti-C3 antibody (solid bar) on D15, RNA was extracted from decidua and placenta on D17 and subjected to real-time PCR analysis for *Il10* and *Tgfb1* transcript levels. *Actb* mRNA was used as an internal control for RNA integrity. Values represent mean ± SEM from three independent experiments, each containing duplicates. Asterisks indicate statistically significant differences in mRNA levels (P < 0.05) when compared to that of the control, PBS treatment.

Alternative hemolytic complement test revealed that goat erythrocyte lysis with the serum obtained from the anti-C3 antibody injected P17 mice was down-regulated, indicating that alternative complement pathway activity against alien substances, in which C3 predominantly involved, was successfully restricted (Fig 6A). To further the role of iC3b on the regulation of anti-inflammatory cytokines, qPCR analysis was also executed in decidual or placental RNAs extracted from those mice injected with the anti-C3 antibody. *Il10* and *Tgfb1* expression was down-regulated in P17 decidua and placenta with the anti-C3 antibody, neutralizing C3, C3b and iC3b (Fig 6B). These results indicated that in addition to iC3b receptor CR3 expression and cellular localization in the spongiotrophoblast layer (Fig 4B), iC3b was a likely complement factor, which regulates the expression of anti-inflammatory cytokines, IL10 and TGFB1, in the placenta during late pregnancy.

## Discussion

Increase in decidual and placental C3, C3b, and iC3b toward the latter part of pregnancy in mice indicate that complement systems from middle to late pregnancy are likely functioning. iC3b is known as an opsonin for the innate immune system and binds the complement receptor, CR3, composed of CD11b and CD18, on the surface of granulocytes, monocytes, NK cells, T and B lymphocytes, causing the enhancement of phagocytosis [26, 27]. In this study, while iC3b receptor transcripts, *Cd11b* and *Cd18*, were highly expressed in P19 placenta, CD11b was localized in the spongiotrophoblast layer. Although a type of immune cells in the spongiotrophoblast layer was not determined, specific CD11b staining was evident in that layer of the placenta. Together, our observations point to the notion that iC3b in the decidua and placenta may act on immune cells in the spongiotrophoblast layer through its binding to CR3, resulting in the induction of anti-inflammatory cytokine expression at the fetal-maternal interface.

In this study, anti-inflammatory *Il10* and *Tgfb1* levels were high in P19 decidual and placental tissues whereas the expression of pro-inflammatory *Il12*, composed of *Il12a* and *Il12b* heterodimers, decreased in P15 and P19 decidua and placenta. These results agree with the previous observation, in which the binding of iC3b to CR3 up-regulates the expression of anti-inflammatory cytokines, *IL10* and *TGFB*, but down-regulates the pro-inflammatory cytokine, *IL12*, resulting in iC3b’s ability to induce the tolerance in the anterior chamber of the eye, an immune-privileged site [15]. Moreover, these anti-inflammatory cytokine mRNA levels in P17 placenta were down-regulated by a single injection of anti-C3 antibody, neutralizing C3, C3b, and iC3b, on P15. Further, the observation in which goat erythrocyte lysis was partially restricted with the serum obtained from P17 mice injected with the anti-C3 antibody. These results indicated that alternative complement pathway through C3 complement system was restricted with the use of anti-C3 antibody, and suggest that iC3b possibly play a role of immunotolerance during the late pregnancy.

High expression of iC3b as well as *Cfi* and *Crry*, required for C3b conversion to iC3b, was found in P11, P15, and P19 deciduas and placentas, whereas the expression of *Cfb* and *Cfd*, required for C3b up-regulation, was not found in any of these samples examined (results not shown). In *Crry* gene ablation study, the percentage of homozygous mutants declined progressively from P10.5 onward because of complement deposition and concomitant placenta inflammation [28]. Moreover, *Crry*-deficient mice exhibited positive staining for activated C3b, and embryonic lethality present in these mice was completely rescued when they were bred to C3-deficient mice [29]. Together with our observations, up-regulation of iC3b from middle to late pregnancy, suggest that the conversion of excessive C3b to iC3b, mediated by CR1 (CD35), MCP, CFH and CRRY [13, 14], is required for the reduction in complement deposition and possible placental inflammation during the peri-parturition periods.

It has been established that the uterus and cervix undergo involution within 30 days of parturition, which involves significant tissue remodeling and turnover [30]. The damaged uterus during or immediately after parturition causes hepatocytes to release acute phase proteins that help to promote tissue repair following the inflammatory process [31]. It is known that complement systems participate in hemostasis and wound healing at sites of tissue injury [32, 33]. Similarly, a separate study reported that C3 cleavage products are required for normal liver regeneration [34]. In this study, high iC3b expression level was found in placenta and decidua during the latter phase of pregnancy just prior to parturition. Although the role iC3b plays in late pregnancy and/or parturition has not yet been fully characterized, iC3b associated up-regulation of anti-inflammatory cytokines during late pregnancy suggests that iC3b may function in the maintenance of delicate balance between pro- and anti-inflammatory environments. This balance may lead to ease of endometrial tissue repair processes following parturition in a manner similar to tissue regeneration in other organs.

Evidence accumulated indicates that complement dysregulation is associated with serious pregnancy complications, resulting from insufficient placentation. For example, patients with placental abruption exhibit a disordered activity of complement system, especially that of C3-activator [35]. Following placental injury, increased C3b deposition has been observed in patients with antiphospholipid syndrome [36]. Moreover, activated complement pathways are found in preeclampsia, suggesting that inhibition of excessive complement activation may be a promising therapeutic approach in the management of preeclampsia [37]. Together, these observations indicate that management of complement expression, especially those derived from C3, is important, particularly the latter part of pregnancy, and suggest that complement systems could be a potential therapeutic target for the treatment of pregnancy disorders originating from complement dysregulation.

In conclusion, the results from this study provide evidence that increased iC3b and its receptor exist at the fetal-maternal interface, and suggest that iC3b may play a role in the regulation of the anti-inflammatory cytokines, IL10 and TGFB1, during the late pregnant period.

## Supporting information

S1 Table Oligonucleotide primers for real-time PCR analyses. (XLSX)

S2 Table Lists of proteins from MALDI-TOF/MS analysis. (XLSX)

S3 Table NC3Rs ARRIVE guidelines checklist. (PDF)
